# A novel classification of subaxial cervical hemivertebrae and associated surgical management

**DOI:** 10.3389/fsurg.2023.1123397

**Published:** 2023-03-17

**Authors:** Jinhui Wu, Miao Hu, Zhengbo Tao, Xin Zhou, Heng Jiang, Tao Lin, Jun Ma, Rui Gao, Ce Wang, Xuhui Zhou

**Affiliations:** ^1^ Department of Orthopedics, Changzheng Hospital, Shanghai, China; ^2^ Department of Orthopedics, Shanghai General Hospital, Shanghai, China

**Keywords:** cervical deformity, hemivertebra, surgical method, classification, reliability study

## Abstract

**Objective:**

To propose and validate a new classification of surgical methods for patients with subaxial cervical hemivertebrae.

**Method:**

This article reviewed cases diagnosed with subaxial cervical hemivertebrae in our hospital from January 2008 to December 2019. The results of preoperative (initial visit), postoperative and/or final follow-up were assessed using the Japanese Orthopaedic Association (JOA) score, Neck Disability Index (NDI) score, spinal balance parameters, and Scoliosis Research Society-22 Questionnaire (SRS-22). We also performed a reliability study to assess this classification.

**Result:**

The classification includes three types. Each type can be divided into two subtypes, and a preliminary algorithm is proposed. Type I: There is an obvious appearance deformity in the neck, there are hemivertebrae in the cervical spine, and only a single hemivertebra of the subaxial cervical hemivertebra needs to be resected. Type II: There is an obvious appearance deformity in the neck, there are hemivertebrae in the cervical spine, and multiple subaxial cervical hemivertebrae need to be removed. Type III: No apparent deformity in the neck, at least one subaxial cervical hemivertebra existed or Klipper-Feil syndrome. Each type is divided into two subtypes, A and B, according to whether the upper and lower adjacent vertebral bodies of the rescected hemivertebra(e) are fused. We propose corresponding treatment methods for different types. We included a total of 121 patients and reviewed the prognosis for each type of patient. All patients achieved satisfactory results. The reliability study showed that the mean interobserver agreement was 91.8% (89.3%–93.4%), and the *κ* value was 0.845 (0.800–0.875). The intraobserver agreement ranged from 93.4% to 97.5%, with a mean *κ* value of 0.929 (0.881 to 0.954).

**Conclusion:**

In our study, we proposed and validated a new classification of subaxial cervical hemivertebrae and proposed corresponding treatment plans for different classifications.

## Introduction

Cervical hemivertebra is caused by congenital defects in the formation of vertebral bodies. Parents with cervical hemivertebra generally present with appearance deformities such as torticollis, facial asymmetry, asymmetric eyes or high and low shoulders (1, 2). Goldstein's reported (3) that the incidence of hemivertebra was 0.33 cases in 1,000 infants diagnosed by prenatal ultrasound. The natural history of cervical hemivertebrae is still unknown, but congenital scoliosis associated with a single segment of the thoracolumbar hemivertebra progresses 1–3.5° annually before puberty (4, 5). Since conservative treatment of this congenital deformity cannot prevent its continued progression, resection of the cervical hemivertebra is a reasonable option. Indications for surgery are radiographic progression of deformity or severe cosmetic deformities (6).

The combined anterior-posterior (A-P) approach for cervical hemivertebra is popular due to the vertebral artery running through the transverse foramen of the cervical vertebra, and sometimes the anterior-posterior-anterior (A-P-A) approach was applied. Wang (7) et al. reported 2 cases of C3 hemivertebra treated with the A-P-A approach, and the deformity was well controlled. Zhuang (8) et al. reported one patient of three hemivertebrae on the same side of C4–6 treated with A-P approach. Michael Ruf (9) et al. reported one case of C2 hemivertebra, in which the A-P approach achieved satisfactory outcome. Yu (10) et al. reviewed 16 cases of cervical hemivertebrae and performed hemivertebra resection and correction through A-P approaches with good results. However, the cases reported cannot be accurately compared due to the difference in deformity location and complexity. So far, no clinical classification system for cervical hemivertebra has been proposed.

Considering this dilemma, we propose a new classification system for subaxial cervical hemivertebrae. Our aim is to establish a new and comprehensive classification that can better describe this disease and guide the surgical plan. We also reviewed cases treated in our hospital and conducted a reliability study to evaluate our new classification.

## Methods and materials

We reviewed patients diagnosed with subaxial cervical hemivertebra(e) in our hospital from January 2008 to December 2019.

The following inclusion criteria were used: (1) At least one hemivertebra is located in the subaxial cervical area (C3–7); (2) At least one segment of subaxial cervical hemivertebra needs to be resected to correct the deformity; (3) Complete preoperative and postoperative examinations (anterior and posterior cervical x-ray films, standing full frontal and lateral spine radiographs, cervical spine CT + 3D reconstruction, vertebral artery CT) in surgically treated patients. Nonsurgical patients need to have at least 2 consecutive years of x-ray films, CT and cervical spine MRI; (4) All patients were followed up for at least 2 years. Exclusion criteria: (1) History of cervical spine surgery; (2) Cervical deformity due to neurofibromatosis, atlantoaxial joint deformity and other sagittal plane imbalance diseases; (3) No subaxial cervical hemivertebra(e) resection during the operation.

The indication for surgery is severe cosmetic deformity and progression of the deformity. Patients treated nonoperatively were also regularly followed up.

### Clinical evaluation

We collected and evaluated relevant clinical data of eligible patients. Age, sex, height, weight, blood loss (BL), operative time (OT), number of fusion segments, follow-up time, cervical spine Japanese Orthopaedic Association Assessment of Treatment (JOA) score, Neck Disability Index (NDI) score, and Scoliosis Research Society-22 Questionnaire (SRS-22) score were assessed preoperatively and 2 years postoperatively.

### Imaging evaluation

Imaging parameters were measured by two surgeons who were blinded between themselves before surgery, immediately after surgery, and 2 years after surgery. The mean value of the following measurement was recorded: (1) Local scoliosis (LS): coronal main curve Cobb angle; (2) Distal compensation curve (DCC); (3) Segmental kyphosis (SK); (4) T1 tilt (T1T): the angle between the line passing through the upper end plate of T1 and the horizontal line; (5) Clavicular angle (CA): the angle between the tangent line connecting the two highest points of the clavicle and the horizontal line; (6) Neck tilt (NT): the angle between the longitudinal axis (the line drawn from the centre of the C2 odontoid process and the centre of the C7 vertebral body) and the longitudinal vertical line; (7) Head tilt (HT): the angle from the middle of the mandible to the mid-perpendicular line of the sacrum; (8) Shoulder balance (SB): the angle between the line connecting the lateral sides of the clavicle and the horizontal line.

### Reliability study

Preoperative imaging data for all patients were retrieved from the Case Archives and Communication System (PACS) by independent observers. All patients were classified twice according to our new classification by three experienced spine surgeons independent of our institution. Intra- and interobserver data were calculated for each component using Kappa and SAS software from Fleiss (SAS Institute, Cary, NC).

### Statistical analysis

Statistical analyses were performed using SPSS 19.0 software (SPSS Inc., Chicago, IL). The measurement data that conformed to a normal distribution and the homogeneity of variances were tested by matched T-test, and the data that did not conform to a normal distribution were tested by the Mann‒Whitney-Wilcoxon nonparametric test. The count data were tested by the chi-square test. Of note, data distribution was determined by using shapiro-wilk test as well as levene's test. Statistical significance was defined as *P* < 0.05. Inter- and intraobserver agreement was determined using Fleiss' *κ* coefficient. *κ* ≤ 0.2 was considered very weak agreement; 0.2 < *κ* ≤ 0.4 was considered weak agreement; 0.4 < *κ* ≤ 0.60 was considered moderate agreement; 0.6 < *κ* ≤ 0.80 was strong agreement; and 0.8 < *κ* ≤ 1.00 was considered to be almost identical or very strong agreement.

### Ethics statement

This study was approved by the ethics committee of our hospital. We reached consensus with all participants, and all processes were carried out in accordance with the Declaration of Helsinki and relevant Chinese policies.

## Result

### A new classification and case review

**Figure 1:**
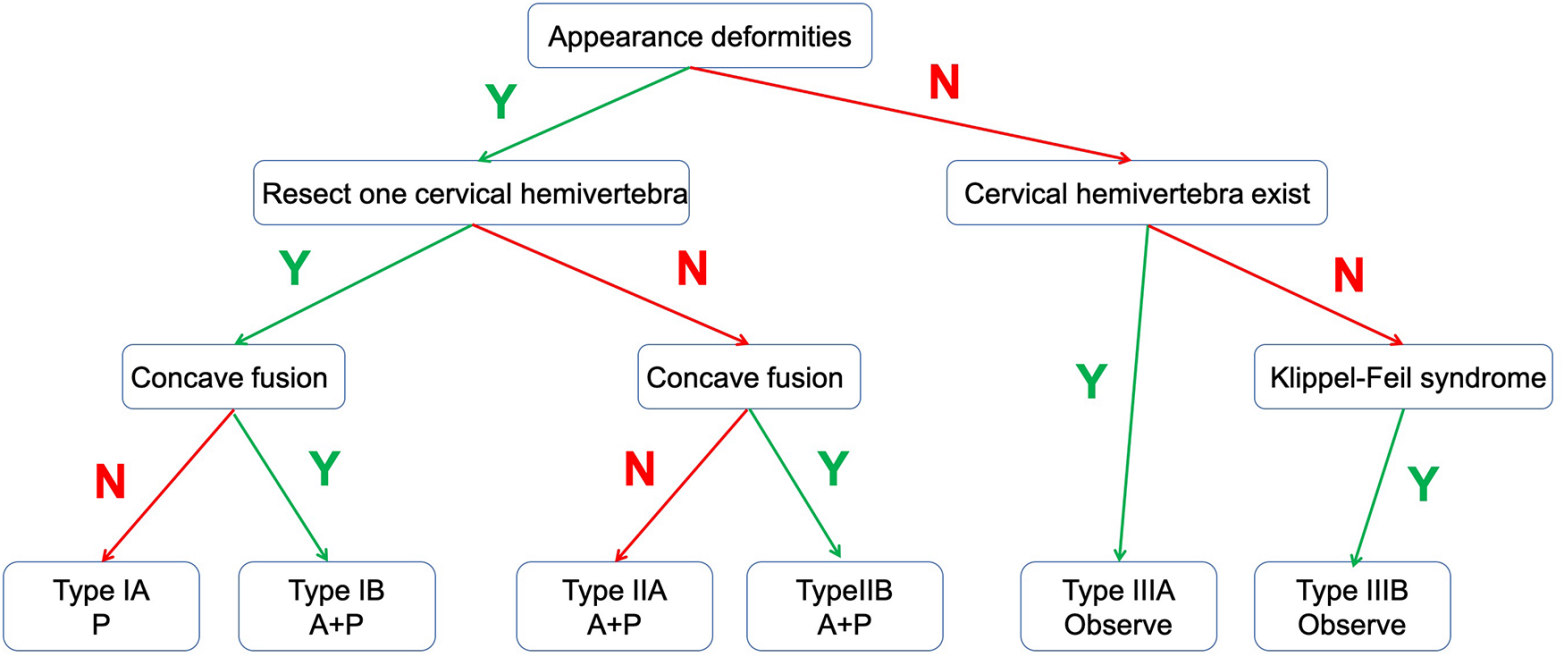
Algorithm for classification of subaxial cervical hemivertebra. Y, YES; N, NO.

**Figure 2:**
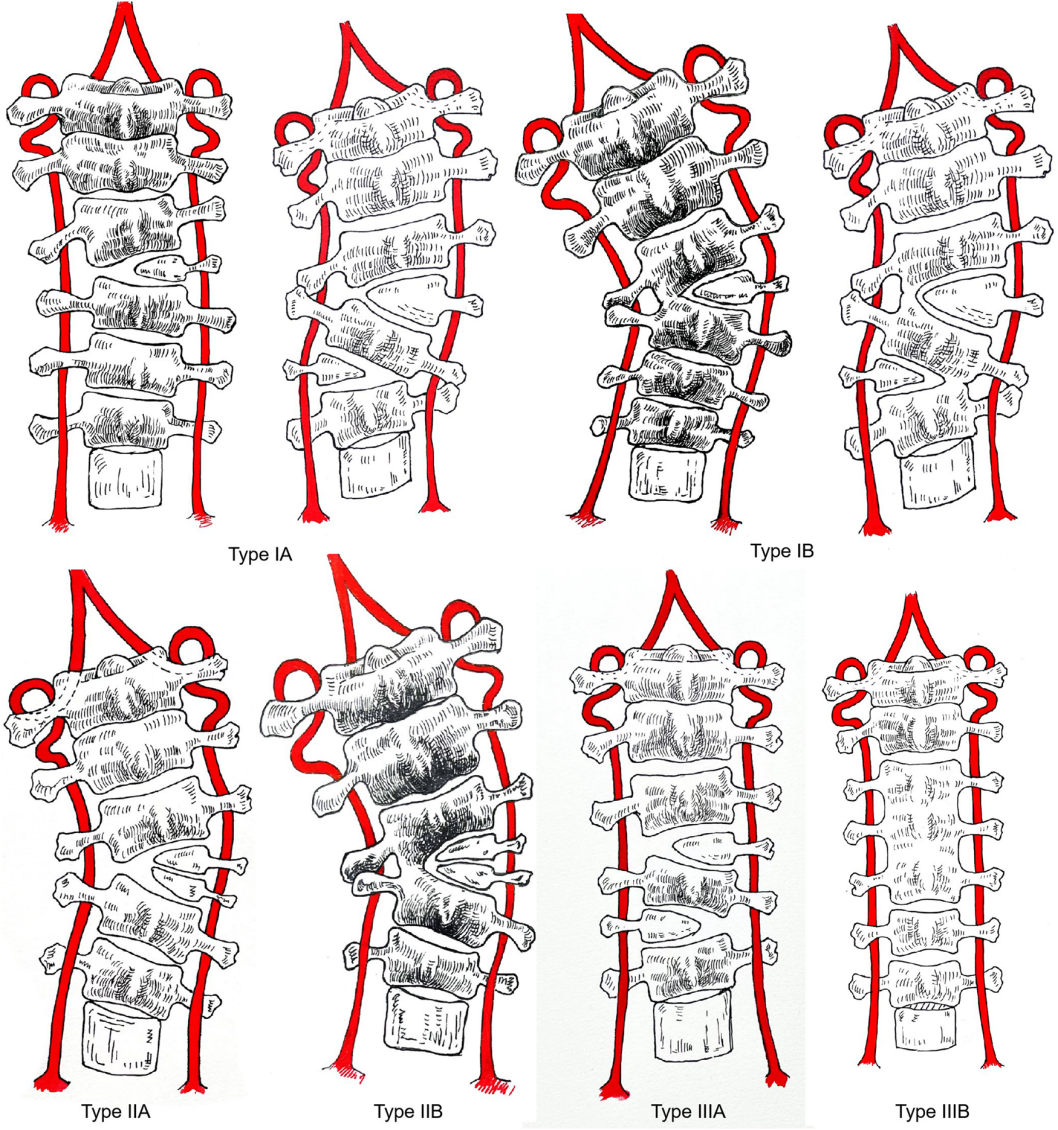
Classification of subaxial cervical hemivertebrae. Type IA: There is an obvious appearance deformity in the neck, there is a hemivertebra in the subaxial cervical and only one hemivertebra of the subaxial cervical needs to be resected to rectify the balance of the cervical spine, and the vertebra at the distal and proximal ends of the hemivertebra to be resected are not fused on the concave side. Type IB: There is an obvious appearance deformity in the neck, there is a hemivertebra in the subaxial cervical and only one hemivertebra of the subaxial cervical needs to be removed, and the vertebra at the distal and proximal ends of the hemivertebra to be resected are fused on the concave side. Type IIA: Obvious appearance deformity in the neck, there are hemivertebrae in the cervical and multiple cervical hemivertebrae need to be resected, and the distal and proximal vertebral bodies of the hemivertebrae to be resected are not fused on the concave side. Type IIB: There is an obvious appearance deformity in the neck, and there are hemivertebrae in the cervical spine. Multiple subaxial cervical hemivertebrae need to be resected, and the distal and proximal vertebral bodies of the hemivertebrae to be resected are fused on the concave side. Type IIIA: There is no obvious appearance deformity in the neck, and there are hemivertebrae in the cervical spine. IIIB: No apparent deformity of the neck or Klipper-Feil syndrome.

We divided these patients into 3 types according to the following questions, and each type was further divided into 2 subtypes: (1) whether there was severe cosmetic deformity; (2) whether a single subaxial cervical hemivertebra or multiple cervical hemivertebrae needed to be resected; and (3) whether the two vertebral bodies proximal and distal to the resected hemivertebra were fused on the concave side (Figures 1, 2).

Type I: There is severe cosmetic deformity in the neck and (a) hemivertebra(e) in the subaxial cervical vertebra, and only one hemivertebra of the subaxial cervical vertebra needs to be resected to restore the balance of the cervical spine. Type IA refers to the deformity without fusion on the concave side and type IB with the two vertebral bodies proximal and distal to the resected hemivertebra fused on the concave side. Treatment strategy: We recommend posterior-only approach for resection of the hemivertebra and correction. For type IB, the hemivertebra should be resected using the anterior approach firstly. And the concave fused vertebra needs to be disconnected. Then, the residual part of the hemivertebra is resected and corrected through the posterior approach.

**Figure 3:**
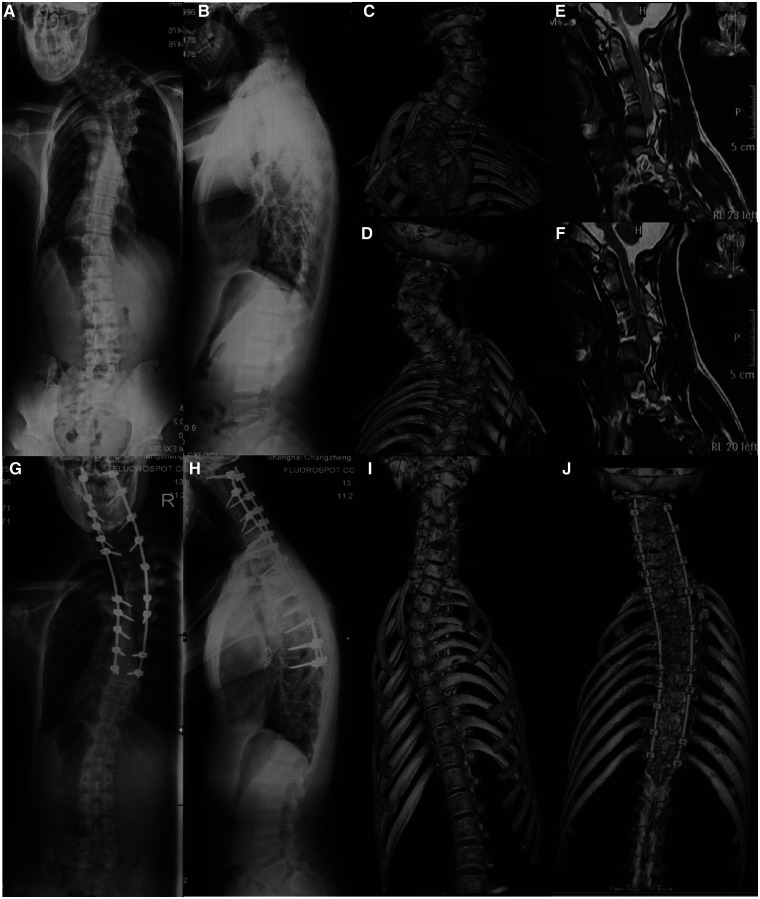
A case of Type IA cervical hemivertebra, male, 18 years old. Figures (**A–F**): C4 hemivertebrae, and C3 and C5 are not fused on the concave side. Figures (**G–J**): simple posterior resection of C4 hemivertebrae, C2-T9 fixation and fusion, 2-year follow-up.

Surgical procedure for Type IA: All of the Surgeries were performed under neuromonitoring of motor evoked potentials (MEPs) and somatosensory evoked potentials (SSEPs). All operations were performed by the same surgeron who was familiar with cervical hemivertebra surgery. The patient was placed in the prone position, and a standard midline incision was made at the back of the neck. The spinous process and lamina were carefully exposed, and the position of the hemivertebra was determined by fluoroscopy. The pedicle screw or lateral mass screw was placed at fusion segments. To increase the accuracy of internal fixation placement, all screws were placed under navigation. The posterior structures of the hemivertebra were subsequently removed, including the lamina, facet joints, and transverse process. The pedicle was then exposed, and the lateral cortex of the hemivertebra was exposed by blunt dissection to avoid damage to other tissues on the ventral side. A temporary rod was placed on the concave side to stabilize the spine before the vertebral body was removed. Next, the hemivertebra and superior and inferior intervertebral discs including the cartilaginous endplates were completely resected through the hemivertebra pedicle. The screws were connected on both sides with connecting rods, and the space was closed after excision of the hemivertebra through concave distraction and convexity compression until it was completely closed. If the hemivertebral body was too large and the gap was difficult to close by instrumentation, the autologous iliac bone was applied for anterior column reconstruction. Care was taken to ensure that the nerve roots and dural sac were not compressed. After correction, a large amount of bone grafting was performed in the posterior column. Besides MEPs and SSEPs, arousal tests were routinely performed prior to shutdown. The incision was closed layer by layer after rinsing (Figure 3).

**Figure 4:**
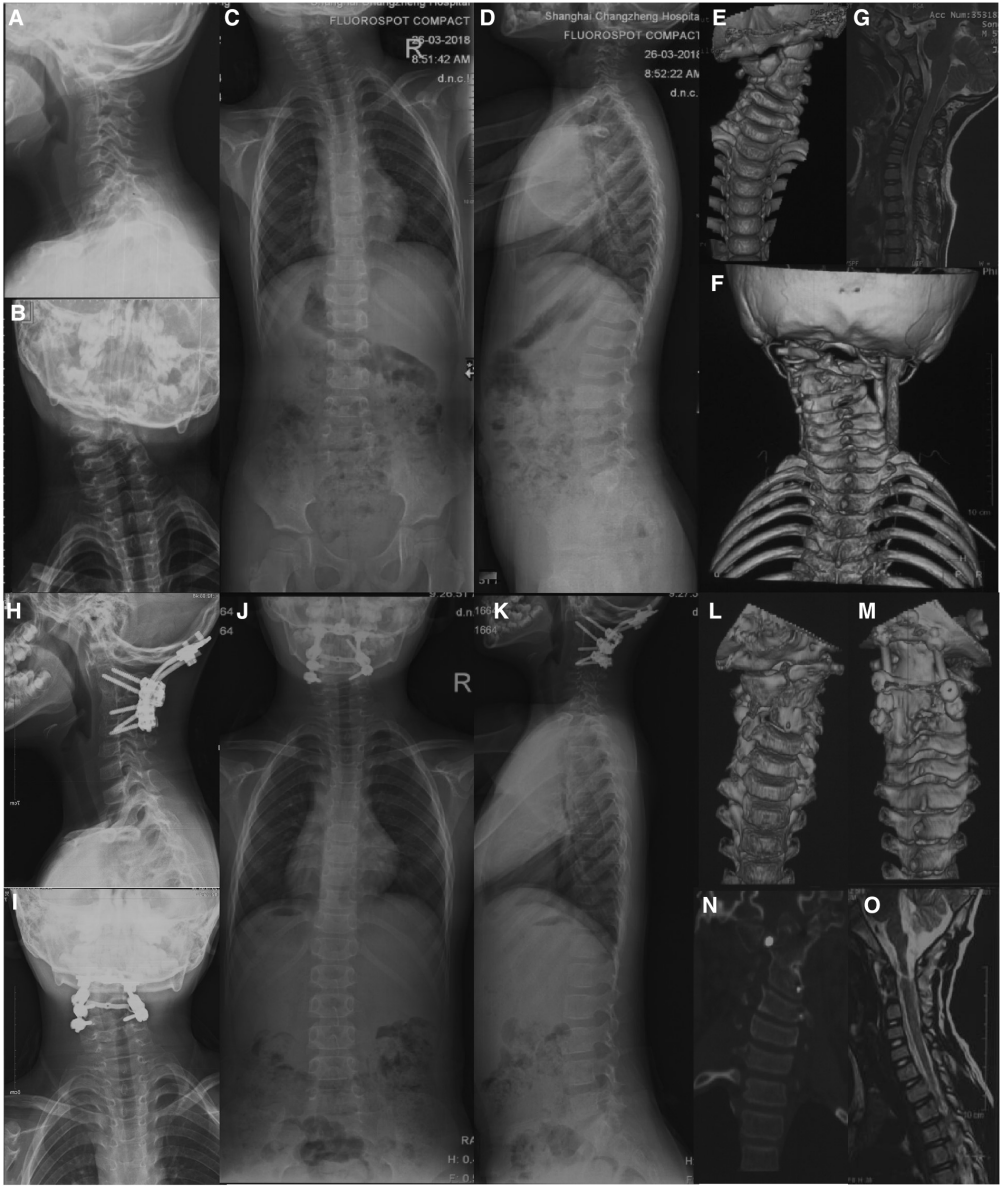
A case of Type IB cervical hemivertebra, male, 6 years old. Figures (**A–G**): C3 hemivertebrae with fusion of C2 and C4 on the concave side; Figures (**H–O**): A-P approach resection of C3 hemivertebrae, occipital-cervical fixation and fusion, and 2-year follow-up.

Surgical procedure for Type IB: The patient was placed in the supine position, and the Robinson-Smith approach was used to expose the cervical hemivertebra. The longus cervicalis covering the lateral side of the hemivertebra was excised. The nerve hook was used to identify the transverse foramen. The anterior wall of the vertebral artery and the lateral wall of the hemivertebra were exposed, and the vertebral artery was freed and protected. And then the entire hemivertebral body, including the V-shaped intervertebral disc above and below it, was removed with an ultrasonic osteotome. Excision was performed until the pedicle was exposed (the hemivertebra with incomplete segments was cut off directly with an ultrasonic osteotome on the side fused with the adjacent segment in the same way). Then, the longus neck muscle covering the ventral side of the fusion vertebral body on the concave side was cut off. The outer edge of the fused vertebral body and the corresponding transverse foramen were explored, and the fusion site was gradually released and disconnected with an ultrasonic osteotome until the outer walls of the two vertebral bodies were completely separated. During the excision process, attention was given to protect the spinal cord and nerve roots and avoid excessive stretching. The anterior surgery was terminated when the pedicle was excised to the level of the nerve root. The wound was drained and then sutured layer by layer and the patient was changed to the prone position. In the prone position, after incision and exposure of the hemivertebra as mentioned above, the laminae of the hemivertebra were excised with an ultrasonic osteotome and cavity forceps to expose the spinal cord. Then, the lateral mass, facet joint of the hemivertebra, and adjacent facet joints were polished with a drill and excised. The nerve groove was used to explore the posterior wall of the transverse foramen. After resection, the vertebral artery was completely freed, and the pedicle of the hemivertebra was gradually excised to the ventral side until the defect in the anterior approach merged. The pedicle screws on both sides were connected with connecting rods and the gap was slowly closed after the hemivertebra was removed by concave distraction and convexity compression until it was completely closed. If the dural sac was severely folded, a portion of the lamina adjacent to the vertebral was removed to avoid compression of the spinal cord. After correction, the posterior structures were decortified, and the cancellous bone in the resected hemivertebra was trimmed for posterolateral fusion (Figure 4).

A total of 36 patients were classified as type I, of which 21 were type IA and 15 were type IB, and all of them underwent the above surgery. The mean preoperative age, mean height, mean weight, mean operation time, mean intraoperative BL, mean follow-up time and mean fusion segment of type IA were 11.5 ± 4.4 years, 135.2 ± 20.0 cm, 36.1 ± 8.8 kg, 219.4 ± 38.2 min, 605.2 ± 111.4 ml, 64.1 ± 28.9 months and 7.2 ± 4.0, respectively. The preoperative LS improved from 37.7° ± 4.5° to 12.0° ± 4.2° postoperatively (*P* < 0.001), with an average correction rate of 66.9% ± 14.3%. The DCC was corrected from 18.1° ± 4.0° to 10.8° ± 3.8° (*P* < 0.001). The T1T increased from 17.5° ± 4.7° to 9.7° ± 4.8° (*P* < 0.001). The CA improved from 12.1° ± 4.1° to 7.2° ± 3.8° (*P* < 0.001). The NT increased from 18.8° ± 5.5° to 10.6° ± 5.5° (*P* < 0.001). The HT was corrected from 19.6°±5.4° to 10.5° ± 5.3° (*P* < 0.001). SB was corrected from 6.6° ± 1.9° to 4.0° ± 1.8° (*P* < 0.001). Both the NDI score and SRS-22 appearance score were significantly improved (5.0% ± 3.1%, 4.4 ± 0.1, *P* < 0.05) (Table 1). The mean preoperative age, mean height, mean weight, mean operative time, mean intraoperative BL, mean follow-up time and mean fusion segment of type IB were 12.1 ± 4.1 years, 144.3 ± 22.5 cm, 41.6 ± 8.7 kg, 394.7 ± 48.7 min, 1268.7 ± 182.2 ml, 68.1 ± 26.8 months and 7.4 ± 2.9, respectively. The preoperative LS improved from 33.6° ± 4.0° to 12.9° ± 3.9° (*P* < 0.001), with an average correction rate of 60.2% ± 14.1%. The preoperative DCC angle increased from 17.7° ± 3.9° to 10.1° ± 3.9° (*P* < 0.001). The T1T was corrected from 16.6° ± 4.7° to 9.3° ± 4.6° (*P* < 0.001). The CA was corrected from 10.4° ± 4.2° to 6.2° ± 4.3° (*P* = 0.015). The NT was corrected from 17.1° ± 5.1° to 9.4° ± 5.1° (*P* < 0.001). The HT was corrected from 18.5° ± 5.1° to 8.5° ± 5.0° (*P* < 0.001). SB was corrected from 6.5° ± 1.6° to 4.1° ± 1.6° (*P* < 0.001). The NDI score and SRS-22 appearance score were significantly improved (4.6% ± 3.4%, 4.4 ± 0.1, *P* < 0.001) (Table 2). Nerve root palsy occurred in 5 patients after the operation, including 1 case of C3 palsy, 2 cases of C5 palsy, and 2 cases of C6 palsy. All of these patients had fully recovered by the 1-year follow-up. No vertebral artery injury or dural sac tear was observed during this type of surgery or during follow-up.

**Table 1:** Clinical evaluations in Type IA patients (mean ± SD).

Item	Preoperative	Postoperative	*P* value
Age (years old)	11.5 ± 4.4	—	—
Operation time (min)	—	219.4 ± 38.2	—
Blood Loss (ml)	—	605.2 ± 111.4	—
Mean follow-up time (mos)	—	64.1 ± 28.9	—
Mean height (cm)	135.2 ± 20.0	—	—
Mean weight (kg)	36.1 ± 8.8	—	—
Mean no. of fused levels	—	7.2 ± 4.0	—
**Parameters of spinal alignment**
Local scoliosis (°)	37.7 ± 4.5	12.0 ± 4.2	<0.001
T1 tilt (°)	17.5 ± 4.7	9.7 ± 4.8	<0.001
Clavical angle (°)	12.1 ± 4.1	7.2 ± 3.8	<0.001
Neck tilt (°)	18.8 ± 5.5	10.6 ± 5.5	<0.001
Head tilt (°)	19.6 ± 5.4	10.5 ± 5.3	<0.001
Shoulder balance (°)	6.6 ± 1.9	4.0 ± 1.8	<0.001
Distal compensation curve (°)	18.1 ± 4.0	10.8 ± 3.8	<0.001
Correction rate (%)		66.9 ± 14.3	—
Segmental kyphosis (°)	13.2 ± 4.6	10.9 ± 4.4	0.108
**Clinical outcomes**
JOA Score	15.6 ± 1.1	15.8 ± 1.1	0.577
NDI (%)	10.3 ± 3.4	5.0 ± 3.1	<0.001
**SRS-22 scores**
Appearance	3.0 ± 0.2	4.3 ± 0.2	<0.001
Activity	4.1 ± 0.2	4.2 ± 0.2	0.176
Pain	4.1 ± 0.2	4.2 ± 0.2	0.101
Mental	4.0 ± 0.2	4.1 ± 0.2	0.157
Satisfaction	—	4.4 ± 0.1	—

**Table 2:** Clinical evaluations in Type IB patients (mean ± SD).

Item	Preoperative	Postoperative	*P* value
Age (years old)	12.1 ± 4.1	—	—
Operation time (min)	—	394.7 ± 48.7	—
Blood Loss (ml)	—	1268.7 ± 182.2	—
Mean follow-up time (mos)	—	68.1 ± 26.8	—
Mean height (cm)	144.3 ± 22.5	—	—
Mean weight (kg)	41.6 ± 8.7	—	—
Mean no. of fused levels	—	7.4 ± 2.9	—
**Parameters of spinal alignment**
Local scoliosis (°)	33.6 ± 4.0	12.9 ± 3.9	<0.001
T1 tilt (°)	16.6 ± 4.7	9.3 ± 4.6	<0.001
Clavical angle (°)	10.4 ± 4.2	6.2 ± 4.3	0.015
Neck tilt (°)	17.1 ± 5.1	9.4 ± 5.1	<0.001
Head tilt (°)	18.5 ± 5.1	8.5 ± 5.0	<0.001
Shoulder balance (°)	6.5 ± 1.6	4.1 ± 1.6	<0.001
Distal compensation curve (°)	17.7 ± 3.9	10.1 ± 3.9	<0.001
correction rate (%)		60.2 ± 14.1	—
Segmental kyphosis (°)	12.5 ± 5.1	10.4 ± 4.9	0.27
**Clinical outcomes**
JOA Score	15.7 ± 1.0	15.9 ± 1.1	0.622
NDI (%)	10.3 ± 3.3	4.6 ± 3.4	<0.001
**SRS-22 scores**
Appearance	3.0 ± 0.2	4.2 ± 0.2	<0.001
Activity	4.1 ± 0.1	4.2 ± 0.1	0.112
Pain	4.1 ± 0.2	4.2 ± 0.2	0.269
Mental	4.0 ± 0.1	4.1 ± 0.1	0.591
Satisfaction	—	4.4 ± 0.1	—

Type II: Severe cosmetic deformity in the neck and multiple hemivertebrae of the subaxial cervical vertebrae need to be resected to restore the balance of the cervical spine. They are divided into type IIA and type IIB according to whether the two vertebrae at the proximal and distal ends of the cervical hemivertebrae to be resected are fused on the concave side. The hemivertebrae could be fully segmented, partially segmented or fused with each other. Treatment strategy: For this type, due to the need to resect two or more cervical hemivertebrae, we recommend the combined anterior and posterior approach to remove the hemivertebrae and correct scoliosis.

**Figure 5:**
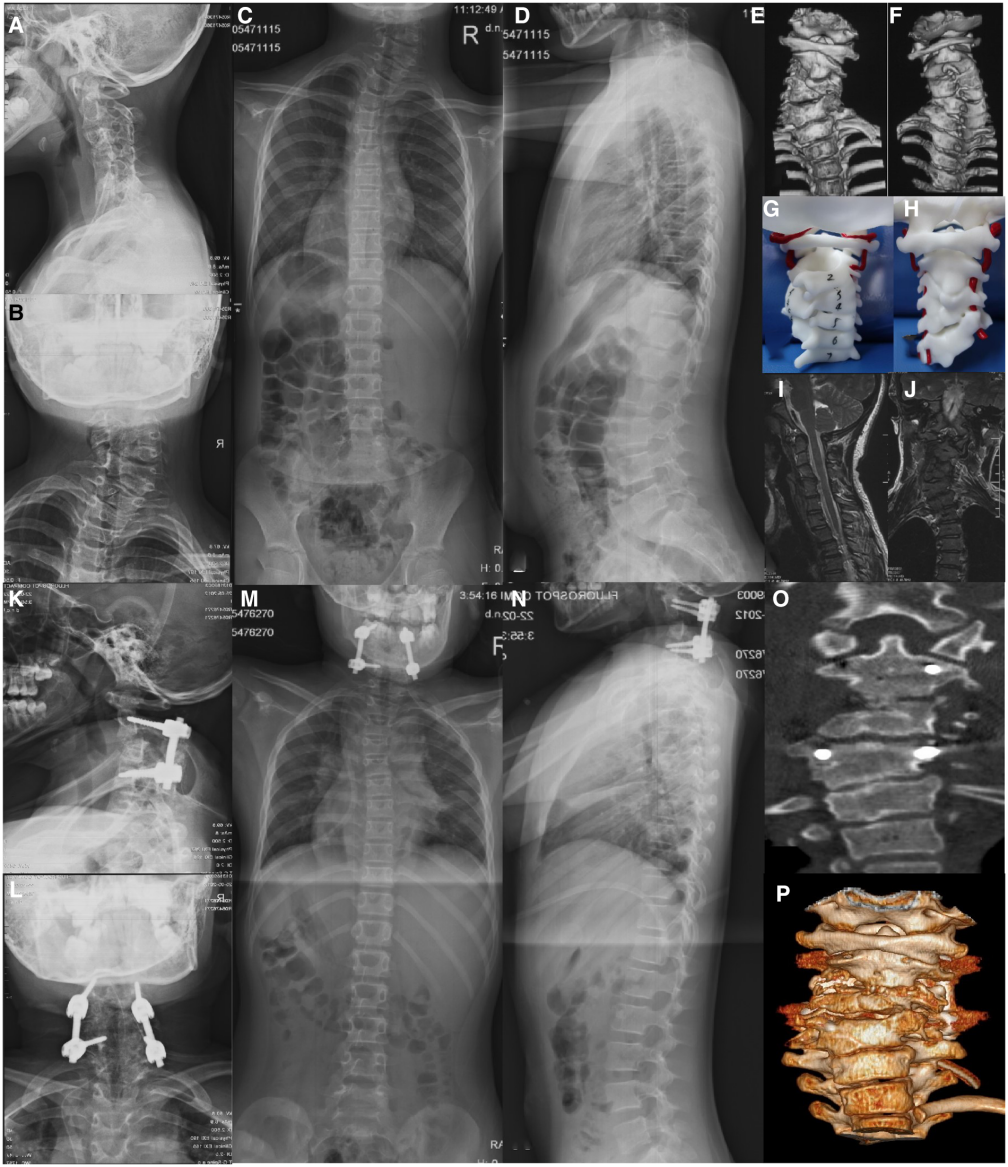
A case of Type IIB cervical hemivertebra, female, 9 years old. (**A–J**): C3, C4 right hemivertebrae, C2 and C5 are fused on the concave side; Figures (**K–P**): A-P approach resection of C3, C4 hemivertebrae, C2-C5 fixed and fused, 2 postoperative year follow-up.

The surgical approach for Type II is similar to that for Type IB. The A-*P* approach is recommended. The difference is that multiple hemivertebrae need to be removed. If it is type IIB, the vertebral body fused on the concave side needs to be disconnected. The rest of the surgical methods are similar to those of type IB (Figure 5).

A total of 9 patients were classified as type II, and all of them underwent the above procedure. The mean preoperative age, mean height, mean weight, mean operation time, mean intraoperative BL, mean follow-up time and mean fusion segment were 11.2 ± 3.6, 137.7 ± 20.7 cm, 34.3 ± 9.6 kg, 409.2 ± 46.6 min, 1279.8 ± 310.2 ml, 75.9 ± 39.3 months and 8.4 ± 3.6, respectively. The preoperative LS improved from 39.4° ± 7.2° to 16.1° ± 5.0° (*P* < 0.001), with an average correction rate of 57.8% ± 15.3%. The preoperative DCC was corrected from 21.1° ± 3.7° to 12.2° ± 3.5° (*P* < 0.001). The T1T increased from 18.2° ± 4.7° to 9.6° ± 4.9° (*P* = 0.002). The CA was corrected from 13.1° ± 2.3° to 7.9° ± 2.4° (*P* < 0.001). The NT was corrected from 22.9° ± 4.1° to 12.3° ± 3.9° (*P* < 0.001). The HT was corrected from 21.1° ± 4.0° to 10.7° ± 4.1° (*P* < 0.001). SB was corrected from 7.8° ± 2.2° to 3.9° ± 2.0° (*P* = 0.002). Both NDI scores and SRS-22 appearance scores were significantly improved (4.7% ± 3.6%, 4.3 ± 0.1, *P* = 0.004) (Table 3). One patient developed C5 nerve root palsy after the operation causing the decreased muscle strength of the upper limb and fully recovered six months after the operation. No vertebral artery injury or dural sac tear was observed during this type of surgery or during follow-up.

**Table 3:** Clinical evaluations in type II patients (mean ± SD).

Item	Preoperative	Postoperative	*P* value
Age (years old)	11.2 ± 3.6	—	—
Operation time (min)	—	409.2 ± 46.6	—
Blood Loss (ml)	—	1279.8 ± 310.2	—
Mean follow-up time (mos)	—	75.9 ± 39.3	—
Mean height (cm)	137.7 ± 20.7	—	—
Mean weight (kg)	34.3 ± 9.6	—	—
Mean no. of fused levels	—	8.4 ± 3.6	—
**Parameters of spinal alignment**
Local scoliosis (°)	39.4 ± 7.2	16.1 ± 5.0	<0.001
T1 tilt (°)	18.2 ± 4.7	9.6 ± 4.9	0.002
Clavical angle (°)	13.1 ± 2.3	7.9 ± 2.4	<0.001
Neck tilt (°)	22.9 ± 4.1	12.3 ± 3.9	<0.001
Head tilt (°)	21.1 ± 4.0	10.7 ± 4.1	<0.001
Shoulder balance (°)	7.8 ± 2.2	3.9 ± 2.0	0.002
Distal compensation curve (°)	21.1 ± 3.7	12.2 ± 3.5	<0.001
Correction rate (%)		57.8 ± 15.3	—
Segmental kyphosis (°)	13.1 ± 6.2	10.2 ± 6.1	0.363
**Clinical outcomes**
JOA Score	15.6 ± 1.0	15.7 ± 1.2	0.837
NDI (%)	10.8 ± 3.6	4.7 ± 3.6	0.004
**SRS-22 scores**
Appearance	3.0 ± 0.2	4.1 ± 0.1	<0.001
Activity	4.2 ± 0.1	4.4 ± 0.1	0.068
Pain	4.1 ± 0.2	4.3 ± 0.2	0.077
Mental	3.9 ± 0.2	4.1 ± 0.2	0.089
Satisfaction	—	4.3 ± 0.1	—

**Figure 6:**
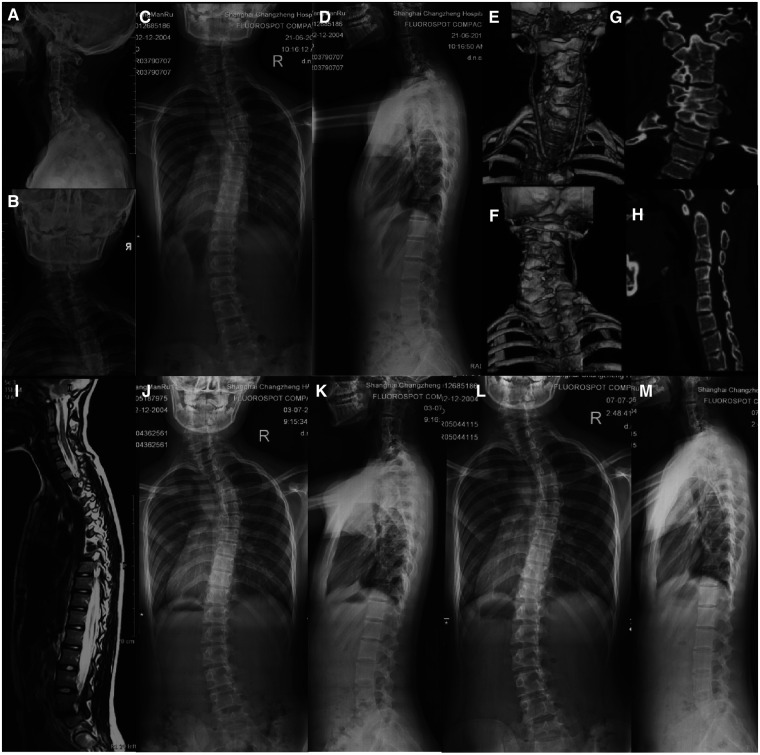
A case of Type IIIA cervical hemivertebra, female, 14 years old. (**A–I**: the left hemivertebra of C4 and the right hemivertebra of C6, with no obvious deformity; Figures (**J,K**): no progression of scoliosis after 1 year; Figures (**L,M**): 2 The scoliosis has not progressed after 2 years.

Type III: No apparent deformity in the neck, and at least one subaxial cervical hemivertebra existed or diagnosis of Klippel-Feil syndrome. According to the position and fusion of the subaxial cervical hemivertebra, type III is divided into type IIIA and type IIIB. Type IIIA refers to the deformity with the presence of the subaxial cervical hemivertebra, and type IIIB refers to Klippel-Feil syndrome. Treatment strategy: This type of cervical hemivertebra could be treated conservatively with regular observation because there is no obvious appearance deformity (Figure 6).

A total of 86 patients were included in this type. But 6 patients showed apparent deformities during follow-up, of which 3 patients were included in type IIB and the other 3 were included in type IB, and all were switched to surgery treatment. The remaining 76 cases were treated conservatively and followed up for more than 2 years. The average height was 135.1 ± 20.8 cm at the initial diagnosis and 157.4 ± 11.5 cm (*P* < 0.001) at the last review. The follow-up time was 79.0 ± 29.0 months. The LS was 14.0° ± 5.1° at the initial diagnosis and 15.6° ± 5.2° (*P* = 0.061) at the last review. The DCC was 8.9° ± 3.0° at the initial diagnosis and 9.6° ± 2.9° (*P* = 0.109) at the last review. The T1T was 10.3° ± 4.3° initially and 11.6° ± 4.3° (*P* = 0.064) at the last re-examination. The CA was 9.8° ± 3.2° at the initial diagnosis and 10.7° ± 3.0° at the last review (*P* = 0.101). The NT was 9.9° ± 3.9° at the initial diagnosis and 11.0° ± 3.9° at the last follow-up (*P* = 0.092). The HT was 9.3° ± 3.6° at the initial diagnosis and 10.4° ± 3.6° at the last follow-up (*P* = 0.085). The SB was 2.6° ± 1.7° at the initial diagnosis and 3.1° ± 1.7° (*P* = 0.117) at the last follow-up. Both the NDI score and SR-S22 appearance score were significantly improved (7.2% ± 1.9%, 3.8 ± 0.2, *P* = 0.108). (Table 4).

**Table 4:** Clinical evaluations in type III patients (mean ± SD).

Item	First visit	Last follow-up	*P* value
Mean follow-up time (mos)	—	79.0 ± 29.0	—
Mean height (cm)	135.1 ± 20.8	157.4 ± 11.5	<0.001
Mean weight (kg)	37.6 ± 8.4	47.1 ± 8.0	<0.001
**Parameters of spinal alignment**
Local scoliosis (°)	14.0 ± 5.1	15.6 ± 5.2	0.061
T1 tilt (°)	10.3 ± 4.3	11.6 ± 4.3	0.064
Clavical angle (°)	9.8 ± 3.2	10.7 ± 3.0	0.101
Neck tilt (°)	9.9 ± 3.9	11.0 ± 3.9	0.092
Head tilt (°)	9.3 ± 3.6	10.4 ± 3.6	0.085
Shoulder balance (°)	2.6 ± 1.7	3.1 ± 1.7	0.117
Distal compensation curve (°)	8.9 ± 3.0	9.6 ± 2.9	0.109
Segmental kyphosis (°)	10.4 ± 4.2	11.4 ± 4.0	0.159
**Clinical outcomes**
JOA Score	15.6 ± 1.1	15.3 ± 1.2	0.13
NDI (%)	6.7 ± 1.9	7.2 ± 1.9	0.108
**SRS-22 scores**
Appearance	3.9 ± 0.2	3.8 ± 0.2	0.109
Activity	4.1 ± 0.2	4.1 ± 0.2	0.11
Pain	4.1 ± 0.2	4.2 ± 0.2	0.118
Mental	4.0 ± 0.1	4.0 ± 0.1	0.283
Satisfaction	—	3.8 ± 0.2	—

### Reliability verification

We used intra- and interobserver reliability analyses to test the accuracy of this classification system, using 3 observers and the data of all of the 121 patients included in the study.

The interobserver agreement was 89.9% (89.3%–93.4%), and the *κ* value was 0.845 (0.800–0.875). Agreement among all three observers was consistent for 107 (88.4%) patients during the first observation period and 98 (81.0%) patients during the second observation period. Nonetheless, at least two observers agreed with the overall classification of 121 (100.0%) patients in the first observation and 120 (99.2%) patients in the second observation. The intraobserver agreement for the overall classification ranged from 93.4% to 97.5%, with a mean *κ* value of 0.929 (0.881 to 0.954) (Tables 5–7).

**Table 5:** Inter-observer reliability of the XX classification.

Observers	Cases in agreement between observers				
	Type I	Type II	Type III	Total (%)	Fleiss’ κ
1 and 2	34	7	67	89.3	0.800
1 and 3	34	9	70	93.4	0.875
2 and 3	34	8	70	92.6	0.86

**Table 6:** Agreement among three observers according to the XX classification.

		All three observers	At least two observers
Novel classification	First observation	107	121
	Second observation	98	120

**Table 7:** Intra-observer reliability of the XX classification.

Observers	Cases in Agreement Between First and Second Observation				
	Type I	Type II	Type III	Total (%)	Fleiss’ κ
1	36	10	67	93.4	0.881
2	36	11	71	97.5	0.954
3	35	10	73	97.5	0.953

## Discussion

In this study, we propose a new classification system for subaxial cervical hemivertebrae to guide surgical treatment. It consists of three types, and each type can be divided into two subtypes. Each subtype has corresponding treatment methods. We reviewed each type of case and observed a significant improvement in clinical and radiological outcomes. A reliability study based on the new subtypes and using three observers was employed. The mean interobserver agreement was 91.8% (89.3%–93.4%), with a kappa value of 0.845 (0.800–0.875). The intraobserver agreement ranged from 93.4% to 97.5%, with a mean *κ* value of 0.929 (0.881 to 0.954).

In addition to the cervical hemivertebra, the hemivertebra of the upper thoracic vertebra can also cause obvious appearance deformities, and cervical hemivertebra is often combined with hemivertebra deformities of the upper thoracic spine. This complex deformity needs to be considered in a balanced manner. For this type of deformities, the main indication for surgery is severe cosmetic deformity. Therefore, the existence of a thoracic hemivertebra cannot be ignored because of its contribution to appearance deformities. The classification algorithm proposed by us is based on whether there is an obvious appearance deformity and the number of cervical hemivertebrae that need to be removed. Whether there is fusion of the vertebral body at the head and tail of the hemivertebra to be resected is an indication for concave side release. Some studies have showed that one-stage only posterior resection and correction was suitable for the hemivertebra of the thoracic and lumbar vertebrae (11, 12). However, cervical hemivertebra is particular with a vertebral artery passing through the transverse foramen of the hemivertebra. Therefore, how to protect the vertebral artery during surgery is difficult in the process of hemivertebra resection. According to our case review, type IA patients can be treated with the same surgical strategy as the thoracolumbar hemivertebra because there is no fusion on the concave side. A posterior-only approach can safely complete the free transverse foramen and transpedicular hemivertebra resection. In our case series of 25 patients underwent posterior-only surgery, the postoperative imaging parameters and patient satisfaction scores showed significant improvement. In patients with type IIA, multiple hemivertebrae need to be resected, and multiple-segment osteotomy performed by the posterior-only approach would cause greater risk of nerve root traction, which would easily cause nerve root injury. Therefore, we recommend an A-P approach. In total, we have 5 type IIA patients whose postoperative imaging performance and patient satisfaction scores greatly improved. In type IB and IIB patients, due to the fusion of the upper and lower segments of the hemivertebra, it is difficult to release the anterior fusion through the posterior approach alone. Thus the A-P approach with resection and correction of the hemivertebra was recommended. In a total of 21 patients in these three categories, postoperative imaging performance and patient satisfaction scores showed improvement as well. For type III patients, conservative treatment can be performed due to the inconspicuous appearance of deformities. For the 76 cases, there was no obvious progression of the deformity after at least 2 years of follow-up.

The choice of surgical approach for cervical hemivertebral resection is still controversial. Ruf (13) reported 3 cases of cervical hemivertebra, of which 2 cases underwent the P-A approach and 1 case the P-A-P approach. In all 45 cases of cervical hemivertebra treated by surgery, we used a posterior-only approach in 25 cases, and an A-P approach for the other 20 cases. We choose the surgical approaches mainly for the following reasons: 1. Compared with the thoracolumbar hemivertebra, the complexity of the subaxial cervical hemivertebra is mainly due to the existence of the vertebral artery. Intraoperative separation of the vertebral artery is required to avoid injury. At present, one-stage posterior resection of the thoracolumbar hemivertebra has become popular (11, 12). Similarly, for the planned resection of a single hemivertebra of the subaxial cervical spine, namely, type IA, a posterior-only approach can complete excision of the hemivertebra and orthopaedic treatment. A total of 25 patients underwent posterior-only surgery, and satisfactory outcome was obtained. 2. For type IB and type IIB cases where there is fusion on the concave side, the concave side is released through the anterior approach.3. Some scholars believe that the gap of anterior resection is difficult to completely close by the posterior approach, so anterior surgery is used for fusion as well. We believe that this is the same as the simple one-stage posterior resection of the thoracolumbar hemivertebra. Bone grafting can be carried out through the incompletely closed space using the posterior approach. Complete fusion can also be achieved using a posterior column bone graft, and there is no need to go through the front path for pure fusion again. Therefore, we hope that this simple and reliable classification method can provide a new direction for the research and treatment of subaxial cervical hemivertebrae. In the field of cervical hemivertebra, many clinical questions remain unanswered, including intraoperative management of vertebral arteries, ideal timing of surgery, and selection of fusion segments. A reliable typing system will help us carefully evaluate the outcomes and look ahead for future treatments.

Three limitations of this study should be noted. First, this is a single-center study combined with a literature review, and the sample size is small, which has certain limitations on our analysis. Second, this is a classification system proposed based on our cases and the literature, which needs further validation. Third, the classification system is determined according to the number of subaxial cervical hemivertebrae that need to be resected. Some of cervical hemivertebra could be complex, and cannot be easily summarized, which affects the accuracy of classification.

## Conclusion

In this study, we proposed and validated a new classification of hemivertebrae of the subaxial cervical region and corresponding treatment plans for each classifications. Through case review and reliability analysis, the classification was proven to be comprehensive and easy to grasp by spine surgeons.

## Data Availability

The original contributions presented in the study are included in the article/Supplementary Material, further inquiries can be directed to the corresponding author/s.
